# Detection of Tetrodotoxin Shellfish Poisoning (TSP) Toxins and Causative Factors in Bivalve Molluscs from the UK

**DOI:** 10.3390/md15090277

**Published:** 2017-08-30

**Authors:** Andrew D. Turner, Monika Dhanji-Rapkova, Lewis Coates, Lesley Bickerstaff, Steve Milligan, Alison O’Neill, Dermot Faulkner, Hugh McEneny, Craig Baker-Austin, David N. Lees, Myriam Algoet

**Affiliations:** 1 Centre for Environment Fisheries and Aquaculture Science (CEFAS), Food Safety Group, Barrack Road, Weymouth, Dorset DT4 8UB, UK; monika.dhanjirapkova@cefas.co.uk (M.D.-R.); Lewis.coates@cefas.co.uk (L.C.); lesley.bickerstaff@cefas.co.uk (L.B.); steve.milligan@cefas.co.uk (S.M.); Alison.oneil@cefas.co.uk (A.O.); Craig.bakeraustin@cefas.oc.uk (C.B.-A.); david.n.lees@cefas.co.uk (D.N.L.); myriam.algoet@cefas.co.uk (M.A.); 2 Agri-Food and Biosciences Institute (AFBI), Marine Biotoxin Unit, Chemical Surveillance Branch, Agri-Food and Biosciences Institute–Stormont, Belfast BT4 3SD, UK; Dermot.Faulkner@afbini.gov.uk (D.F.); Hugh.mcineney@afbi.co.uk (H.M.)

**Keywords:** Tetrodotoxins, bivalve molluscs, HILIC-MS/MS, UK shellfish, Tetrodotoxin Shellfish Poisoning (TSP)

## Abstract

Tetrodotoxins (TTXs) are traditionally associated with the occurrence of tropical Pufferfish Poisoning. In recent years, however, TTXs have been identified in European bivalve mollusc shellfish, resulting in the need to assess prevalence and risk to shellfish consumers. Following the previous identification of TTXs in shellfish from southern England, this study was designed to assess the wider prevalence of TTXs in shellfish from around the coast of the UK. Samples were collected between 2014 and 2016 and subjected to analysis using HILIC-MS/MS. Results showed the continued presence of toxins in shellfish harvested along the coast of southern England, with the maximum concentration of total TTXs reaching 253 µg/kg. TTX accumulation was detected in Pacific oysters (*Crassostrea gigas*), native oysters (*Ostrea edulis*) common mussels (*Mytilus edulis*) and hard clams (*Mercenaria mercenaria*), but not found in cockles (*Cerastoderma edule*), razors (*Ensis* species) or scallops (*Pecten maximus*). Whilst the highest concentrations were quantified in samples harvested during the warmer summer months, TTXs were still evident during the winter. An assessment of the potential causative factors did not reveal any links with the phytoplankton species *Prorocentrum cordatum*, instead highlighting a greater level of risk in areas of shallow, estuarine waters with temperatures above 15 °C.

## 1. Introduction

Tetrodotoxin (TTX) is a highly potent neurotoxin [1] which is known to accumulate in a wide range of marine and amphibious species. TTX is responsible for the highest fatality rate of all marine intoxications, being associated most commonly with accumulation in species of fish from the Tetraodontidae family, such as puffer fish [2]. TTX is a sodium channel blocker binding to receptor site 1 with activity similar to the saxitoxins responsible for Paralytic Shellfish Poisoning (PSP). A minimum lethal dose (MLD) in humans was previously estimated at around 2 mg TTX [3,4]. TTX and a range of TTX analogues together termed Tetrodotoxins (TTXs) (Figure S1) are evidenced to be produced by wide range of bacterial species, including those from the genera of *Vibrio*, *Bacillus*, *Aeromonas*, *Alteromonas*, and *Pseudomonas* [5], although the mechanisms of production are not yet elucidated. On the other hand, certain phytoplankton such as *Alexandrium tamarense* and *Prorocentrum minimum* (*cordatum*) have been suggested as an alternative source [6,7,8]. TTXs have been identified in a wide variety of marine species including fish, worms, echinoderms, crustaceans and octopus [9,10,11,12,13,14].

Human intoxications are widely reported, occurring mostly following consumption of pufferfish. TTXs have also been found in gastropods and bivalve molluscs, including the recent detection in European waters. TTXs have been specifically reported in a gastropod from Portugal [15], in mussels (*Mytilus edulis*) and Pacific oysters from England [16], mussels from Greece [7] and in mussels and oysters from the Netherlands [17]. Consequently, there is strong evidence for the presence of TTXs in bivalve molluscs from diverse geographic regions within European waters. With this discovery, there is the potential risk for TTX accumulation in bivalves which can enter the food chain. With the lack of evidence for the mechanisms of TTX production and uptake into marine species, the uncertainties associated with TTX prevalence and the subsequent risk to shellfish consumers is heightened. Additionally, it is important to note that EU regulations for marine toxins in bivalve molluscs do not currently include TTXs, so there is no statutory need to monitor this potential threat in national Official Control (OC) monitoring programmes, furthering the risk of contaminated shellfish entering the market and exposing shellfish consumers to Tetrodotoxin Shellfish Poisoning (TSP) [18]. Nevertheless, controls were introduced by the Netherlands in 2016 where a maximum limit of 20 µg/kg shellfish was initially implemented. This limit was chosen as it equates to the limit of quantitation (LOQ) of the Dutch method [17]. Currently the only data published concerning the occurrence of TTXs in bivalve molluscs from the UK was reported by [16]. Samples from that study consisted of mussels and Pacific oysters harvested from just two sites located in the South of England. With a high proportion of the samples analysed found to contain detectable levels of toxin, there became a strong need to expand the testing to other geographical regions of the UK to determine the prevalence and both geographical and temporal variability throughout the country.

The main aim of this study was therefore to conduct a screen of selected official control (OC) shellfish tissue samples from around the UK for the presence of TTXs. A full screen of all shellfish samples submitted for OC testing was impossible, given the high numbers of samples involved (>3500 per year) and the limitations on spare resource including suitable instrumentation within our laboratories. Consequently a multi-stage approach was taken; firstly analysis was conducted on a selection of OC samples taken from around the entire coast of the UK, including England, Scotland and Wales, between March and October 2014, to provide preliminary indications of the prevalence of TTX and associated spatial and temporal variability. Secondly, as TTXs seemed to be most prevalent in summer, all OC shellfish samples collected in July 2015 were analysed. Thirdly, samples from selected sites in the south of England were assessed between May 2015 and November 2016 to allow for continuous long-term assessment in the most affected areas. Finally, analysis of samples collected from Northern Ireland was performed during 2016. Shellfish samples were extracted using AOAC 2005.06 as part of the routine official control monitoring programme for PSP and subsequently analysed using the validated method of [18]. Extracts were cleaned up and analysed by LC-MS/MS for the presence of TTX, plus the associated analogues 4-epi-TTX, 5,6,11-trideoxy TTX, 11-nor TTX-6-ol, 5-deoxy TTX, and 4,9-anhydro TTX. Results were used to assess the occurrence and prevalence of TTXs around the coast of the UK, and compared with environmental parameters that may have influenced TTX production and/or mollusc accumulation. The overall aim was to inform the risk assessment and management of this potential threat.

## 2. Results

### 2.1. TTXs in UK Shellfish

#### 2.1.1. England

**Figure 1:**
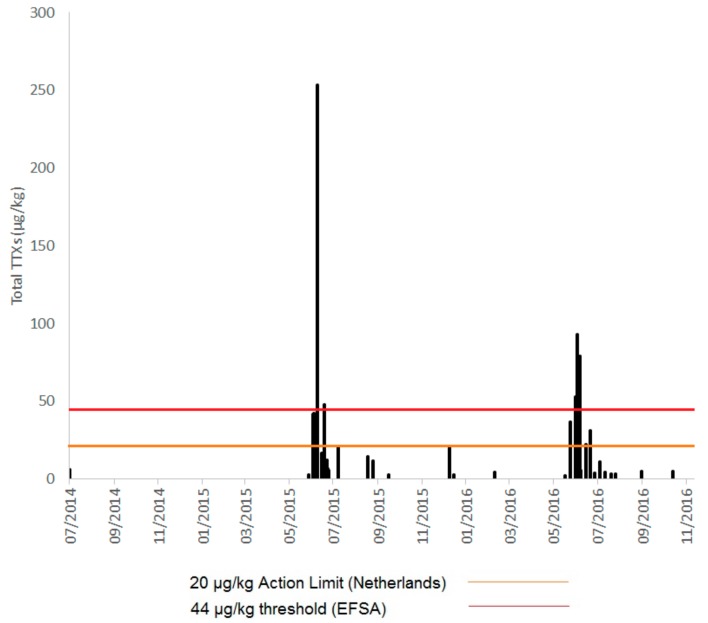
Total Tetrodotoxin (TTX) concentrations (2014–2016) illustrating seasonality of TTX accumulation in bivalve molluscs from Great Britain.

**Figure 2:**
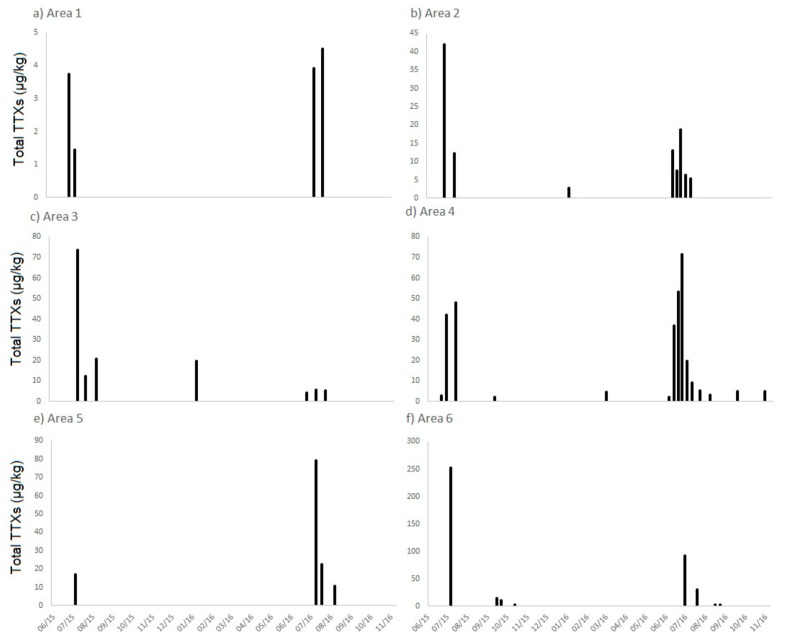
Total TTX concentrations in shellfish from six geographical areas in southern England between May 2015 and November 2016.

Samples tested in the study from England consisted of mussels (*Mytilus edulis*), Pacific oysters (*Crassostrea gigas*), native oysters (*Ostrea edulis*), cockles (*Cerastoderma edule*), hard clams (*Mercenaria mercenaria*), surf clams (*Spisula solida*) and king scallops (*Pecten maximus*), both whole and pre-processed (adductor and roe only). These had been collected across England during the warmer months of July to October 2014 and from southern England during May to October 2015. In addition, samples from six selected areas in southern England, found to contain the highest prevalence for TTX-positive shellfish during 2014–2015, continued to be monitored after October 2015 to obtain a long-term assessment spanning 1.5 years (May 2015–November 2016). Of the 477 samples collected from shellfish in classified production areas from England between 2014 and 2016 and tested in this study, TTXs were detected in 55 samples (12%) at summed TTX concentrations above the limit of reporting (LOR) of 2 µg/kg. Fourteen samples (3.0% of total samples) were found to contain total TTXs above the current Dutch limit of 20 µg/kg (Figure 1), although an additional two samples were found to contain total TTXs very close to this limit (>19.5 µg/kg). All of these samples were obtained from shellfish harvesting sites along the south coast of England (Figure S2). The highest concentrations of total TTXs reached 253 µg/kg in a Pacific oyster sample collected in July 2015. TTXs were found in winter as well as summer, however total summed concentrations above 20 µg/kg were all detected in June, July and August. One mussel sample, however, collected in January 2016 was found to return a total TTXs value of 19.9 µg/kg (Figure 1). Figure 2 illustrates the seasonality of TTX occurrence in shellfish from each of the six main southern regions (areas 1 to 6 inclusive, with each area consisting of geographically close shellfish harvesting beds) which were subjected to regular testing between May 2015 and November 2016. Whilst differences in total TTX concentrations and longevity of toxicity are evident between the six geographical areas, there are strong indications of pronounced toxicity during the summer months in all six areas.

**Figure 3:**
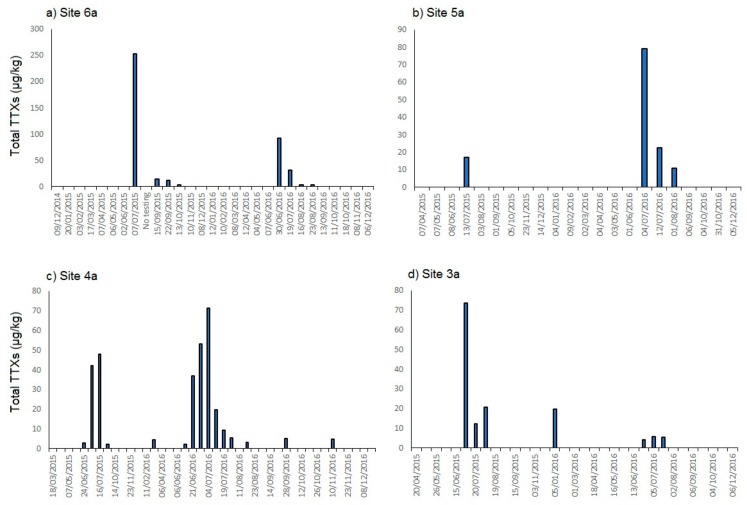
Total TTX concentrations in shellfish from four example shellfish harvesting areas: (**a**) Pacific oysters from site 6a; (**b**) native oysters site 5a; (**c**) hard clams site 4a; and (**d**) mussels site 3a.

In the majority of shellfish harvesting areas found to contain TTX positive samples, the sampling frequency was not high enough to give a good indication of uptake or depuration rates. At the site where the highest toxin concentrations were found in July 2015 (area 6, site a), Pacific oysters are sampled for OC testing on either a fortnightly or monthly basis, depending on the time of year and the presence of regulated marine toxins in OC samples. One month prior to the TTX positive sample, no TTXs were detected, showing there was no prior warning one-month before the high concentration. After the high result, due to a sample collection issue, the next sample available for TTX testing was taken two months later during September. Consequently, there were no useful data describing depuration rates during this period of time (Figure 3a). One harvesting area within area 5 (site 5a; Figure 3b) showed a maximum TTX concentration in native oysters of approximately 80 µg/kg during early July 2016. The next sample taken was two weeks later, which was found to contain around 20 µg/kg, indicating the removal of 75% of the total TTX content within two weeks. The next sample, taken two weeks later, showed a further reduction in close to 50%. Site 4a (Figure 3c) provides the most useful record of uptake and depuration rates of any of the monitored sites, with the sampling of hard clams at this site being conducted fortnightly during summer 2016. TTX was first detected in the clams at concentrations just above LOR on 14 June, with the subsequent sample from 21 June containing 37 µg/kg. For the next two samples, toxin concentrations were measured at 53 and 72 µg/kg, respectively. After this maximum concentration, two weeks later, the levels had reduced by 70% to 20 µg/kg, with the subsequent sample showing a further 50% reduction to approximately 10 µg/kg. It is noted, however, that these tentative patterns are not consistent, with other sites such as 3a (Figure 3d) showing a rapid drop in toxin concentrations over a two week period in mussels, followed by a 40% increase in the subsequent fortnight, before concentrations dropped below LOR.

**Figure 4:**
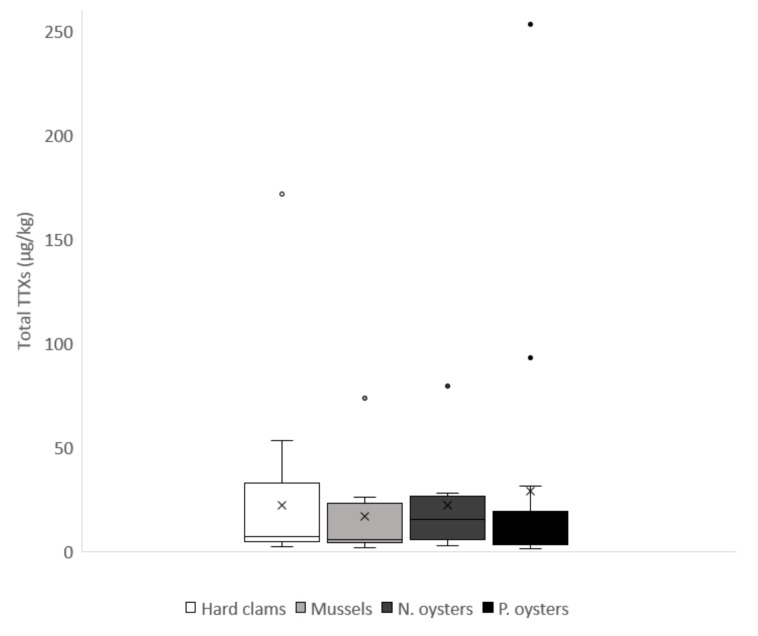
Box and whisker plot illustrating TTX results (µg/kg) quantified >LOR for hard clams (*n* = 24), mussels (*n* = 9), native oysters (*n* = 8) and Pacific oysters (*n* = 16) from England (2014–2016). The box shows the distribution of the data into quartiles, the whiskers show variability outside the upper and lower quartiles, with points outside the whiskers considered outliers. The interquartile line highlights the median, with the X corresponding to the mean.

The toxins were detected in a variety of species including mussels, Pacific oysters, native oysters and hard clams. Between 2014 and 2015, only mussels, Pacific oysters and hard clams were found to contain total TTXs above 20 µg/kg. During 2016, however, samples containing TTXs > 20 µg/kg were found in all four species, with highest concentrations again quantified in Pacific oysters. The mean ± s.d total TTX concentrations were 28.9 ± 64.0, 22.2 ± 24.6, 22.0 ± 35.8 and 17.0 ± 22.8, μg/kg for Pacific oysters, native oysters, hard clams and mussels, respectively. The box and whisker plot illustrates the variability of TTX results obtained in shellfish of each species during the whole study (Figure 4). Only one representative species was collected at each OC monitoring site, therefore a comprehensive evaluation of uptake differences between species was not possible from these data. No TTXs were detected in cockles and surf clams. In addition, all 51 non-OC king scallop samples, collected through the wild *Pectinidae* verification process, were found to be TTXs negative.

#### 2.1.2. Scotland

In total, 670 Scottish samples were collected and tested between March and October 2014 as well as during the month of July 2015. Most of these were the 660 OC samples from routinely monitored classified production areas, with the remainder consisting of 10 verification scallop samples. The vast majority of samples (668 (99.7%)) were not found to contain TTXs. Only two samples showed the presence of TTX above 2 µg/kg and only one sample of mussels collected during July 2014 contained total TTX concentrations above 20 µg/kg (26 µg/kg) (Table 1).

#### 2.1.3. Wales

Twenty-eight shellfish samples were collected from routinely monitored classified shellfish production areas in Wales and tested in this study. These were collected during July to September 2014 and July 2015 and consisted of cockles and mussels only. Following HILIC-MS/MS analysis, none of these samples were found to contain TTX above quantifiable levels (Table 1). TTX was detected at trace levels in only one Welsh sample of cockles, but could not be confirmed at concentrations above LOR (2 µg/kg).

#### 2.1.4. Northern Ireland

Fifty-seven Shellfish samples were collected between May and September 2016, consisting of mussels and Pacific oysters from selected classified shellfish production areas. No TTXs were detected in any of the samples collected and tested in this study (Table 1).

### 2.2. Toxin Profiles

**Figure 5:**
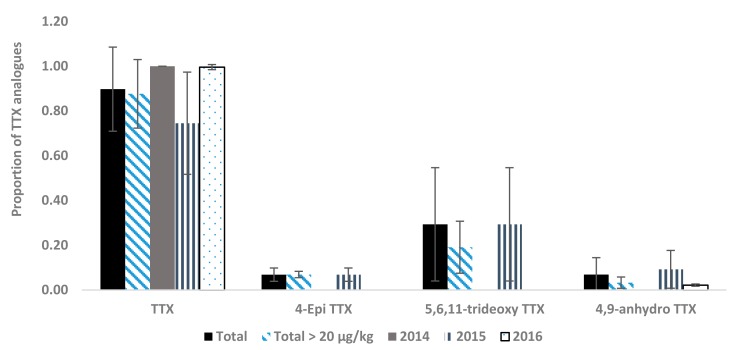
Mean proportions of TTX analogues (±s.d) quantified in UK bivalve molluscs during 2014–2016 (*n* = 57), 2014–2016 and >20 µg/kg (*n* = 15), 2014 only (*n* = 2), 2015 only (*n* = 22) and 2016 only (*n* = 33).

Quantified concentrations of TTX toxins were summed to give the total concentration of TTXs. The relative amount of each individual analogue was subsequently calculated as the proportion of total TTXs, with values used to calculate mean proportions with associated standard deviations (s.d). The dominant TTX analogue found across all the samples quantified was found to be the parent TTX toxin. TTX represented a mean of 90% of the total TTXs concentrations quantified throughout all positive samples from three years in this study. Other analogues detected were 4-epi TTX in samples containing higher total concentrations, 5,6,11-trideoxy TTX and 4,9-anhydro TTX, with mean proportions of 7%, 29% and 7% respectively. In addition, two samples from 2016 were found to contain low concentrations of 5-deoxy TTX. Consequently, the proportions of TTX analogues, whilst dominated by TTX, were found to vary. Figure 5 illustrates the toxin profiles from all samples measured as well as from year to year. Results indicate the near exclusive presence of TTX in positive samples collected during 2014 and 2016, but with 2015 showing higher relative proportions of other analogues, most notably 5,6,11-trideoxy TTX. The total profile calculated only from samples >20 µg/kg showed similar results, thus inferring that the relative proportions of 5,6,11-trideoxy TTX and 4,9-anhydro TTX are not concentration related (Figure 5). Results showed there were no species-related differences in the occurrence of different TTX analogues, with 4-epi TTX, 5,6,11-trideoxy TTX and 4,9-anhydro TTX all detected in clams, oysters and mussels (data not shown). Overall, however, all samples except one containing TTXs were found to contain TTX itself, so there was no evidence for shellfish containing TTXs without the presence of the parent toxin. The one exception was a sample from 2016, which contained very low concentrations of 5-deoxy TTX (2.88 µg/kg).

### 2.3. Links to Environmental Conditions

#### 2.3.1. Water Temperatures

Seawater temperatures recorded by shellfish sampling officers were compared against quantitative HILIC-MS/MS results for the determination of TTXs in the bivalve mollusc samples. Out of the 1222 samples in this study, only 580 (47%) were provided with a water temperature. Nevertheless, with 580 temperature data points, any potential relationship between TTX occurrence and temperature could be assessed. Table 2 shows the results from this comparison and provides an indication that, with a low number of exceptions, TTXs are typically present in shellfish sampled from water temperatures at ≥15 °C. Whilst there is no statistical correlation between these two parameters (correlation coefficient (r^2^) of linear regression between water temperature and total TTX concentrations = 0.02), and there are numerous shellfish samples harvested in water above 15 °C which did not show any presence of TTXs, the 15 °C threshold seems to indicate the limit above which TTXs are more likely to occur in shellfish tissue. The one exception was the mussel sample harvested from the south coast of England, during January 2016, when the water temperature measured 8.4 °C. Unfortunately, the seawater temperature was not recorded for the one mussel sample found to contain TTXs > 20 µg/kg in Scotland, harvested during July 2014.

#### 2.3.2. Water Depth and Salinity

No specific hydrographic parameters were recorded during the shellfish sampling for this study. Consequently, shellfish sites were categorized instead based on their setting, in particular water depth. Specifically, sites were defined as either intertidal/shallow (0–5 m depth), medium depth (5–20 m) or deep water (>20 m). Depth categorization was performed on English sites only, given the absence of any significant toxin levels in shellfish from Wales, Northern Ireland and Scotland. Out of 477 English shellfish samples, 28 (6%) were defined as deep water, 60 (13%) as medium depth, with the remaining 389 (81%) categorized as inter-tidal or shallow water. Table 3 summarises the numbers of TTX-positive samples in each of these three depth-related categories. Results indicate that out of 55 TTX-positive samples, 51 (93%) were obtained from inter-tidal or shallow water environments. The four remaining samples were all harvested from the same medium-depth location.

In the absence of salinity measurements from each monitoring location, sites were additionally categorized as low, medium and high salinity, by defining these as either riverine (138 sites; 29%), estuarine (236 sites; 49%) or open sea (103; 22%). Table 3 also summarises the results obtained from shellfish sites in each of these categories. Forty-five out of the 55 TTX-positive samples (82%) were from estuarine shellfish beds, presumably associated with lower levels of salinity in comparison to open marine waters. Out of the remaining 10 samples, four were collected from the same deep water sites as described above, whilst the six riverine samples were all found to contain only low concentrations of TTXs (<5 µg/kg).

### 2.4. Links to Prorocentrum Cordatum

**Figure 6:**
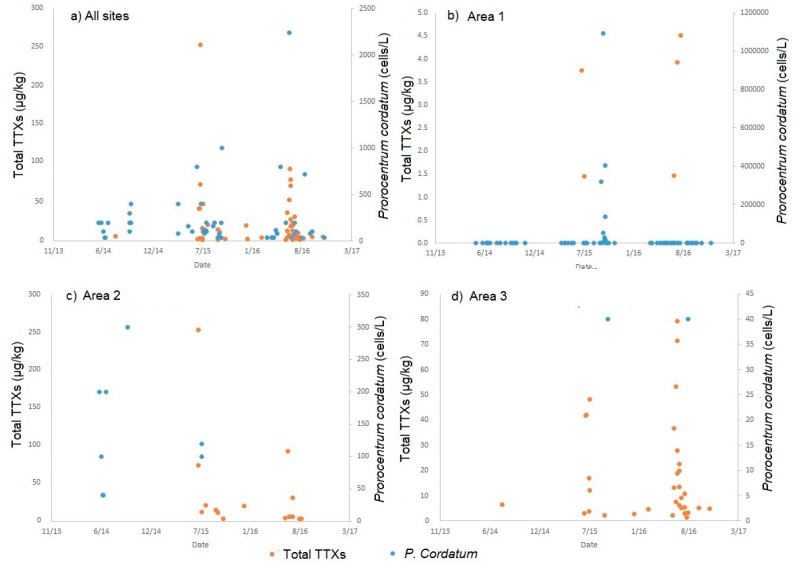
Relationship between the presence of TTXs in bivalve molluscs and *P. cordatum* in the water column: (**a**) all sites combined; (**b**) area 1; (**c**) area 2; and (**d**) area 3.

Figure 6 illustrates the comparison between the detection of *P. cordatum* in the water column and the quantitative levels of TTXs in bivalve molluscs. Results indicate that *P. cordatum* is detected quite commonly in some areas along the southern coast of England, but with variable cell densities from year to year. Looking at all sites together, both the phytoplankton and toxins are present around the same times during 2015 and 2016 in particular. In specific areas, such as area 1, there are no indications of a correlation between the two measurements, with TTX-positive samples during July 2015 and July 2016 not relating to the occurrence of *P. cordatum* which was found at higher cell densities later in the summer of 2015 and at no significant concentrations during the whole of 2016. Similarly, *P. cordatum* was enumerated to cell densities up to 300 cells/L during June 2014, with no TTX detected, whereas during 2015 and 2016 high concentrations of TTXs did not occur with any significant detection of *P. cordatum*. Sites within area 3 showed a large number of shellfish samples containing TTXs through 2015 and 2016 in particular, but with only two positive *P. cordatum* samples recorded at very low cell densities (maximum 40 cells/L). Overall, the highest cell densities of *P. cordatum* were enumerated in the samples taken within area 1, which conversely exhibited the lowest concentrations of TTXs in the shellfish, compared to other geographical areas.

## 3. Discussion

### 3.1. Presence of TTXs in UK Shellfish

The results obtained from this study provide evidence for the presence of Tetrodotoxins in bivalve molluscs sampled as part of the official control biotoxin monitoring programmes of the UK. Previous work from our team had shown the first evidence of TTX accumulation in bivalve molluscs from anywhere in European waters, based upon the results obtained from two specific research monitoring sites along the south coast of England during 2013–2014 [16]. Here, confirmation has been provided for the continued presence of TTX, along the south coast of England in particular, between 2014 and 2016 inclusive, confirming the results of the original study were not a one-off event. As such, this study highlights a continued risk to food consumers from TTXs, noting in particular that these toxins are resistant to degradation under food processing conditions such as cooking or freezing [19]. Overall, the majority of samples analysed contained total TTX concentrations below the 44 µg/kg EFSA guidance threshold [20]. Whilst increasing proportions of TTX-positive results were found from year to year (Table 1), it must be stressed that there were significant differences in the locations of samples obtained for this study each year, most notably with the focus in 2016 on sites along the Southern coast of England previously shown to contain TTX-positive shellfish. From these data alone, it is impossible to determine whether the prevalence of TTX in England is changing over time. A prolonged systematic study will be required to assess this over several years before any conclusions regarding temporal variability in TTX accumulation in bivalve molluscs can be reached. The maximum concentration of 253 µg/kg is substantially higher than the levels reported in our first study [16] and similar to the maximum level of TTXs (223 µg/kg) quantified in molluscs from Greece [7]. Interestingly, to date, the concentrations detected in bivalve molluscs in Europe are of an order of magnitude lower than those quantified in the digestive glands of the trumpet shell *Charonica lampas lampas* harvested from Portugal [15]. In that study, TTX and 5,6,11-trideoxy TTX were quantified by LC-MS at 315 mg/kg and 1004 mg/kg respectively, although follow-up studies in the same species showed evidence for much lower concentrations generally [21,22]. Our current maximum concentration equates to ~12.5 times the limit of 20 µg/kg currently in place within the Netherlands [17]. Our maximum level, however, would result in the ingestion of ~100 µg TTX following the consumption of a 400 g meal portion, which equates to approximately 5% of the MLD of 2 mg TTX [3,4]. Furthermore, seven samples were found to contain total TTX concentrations above the value of 44 µg/kg proposed by EFSA as being the concentration below which no adverse effects would be experienced in human shellfish consumers, assuming a portion size of 400 g [20]. A full and thorough risk assessment is therefore urgently needed to assess the validity of such guidance limits, in order for these results to be placed into context for risk assessment purposes.

TTX concentrations determined at each of the six main geographical areas around the south coast of England show notable spatial differences in toxin accumulation. The reasons for this could not be determined from this study alone, particularly in the absence of evidence for toxin production and uptake mechanisms. They are, however, likely to result from differences in localised environmental factors as well as the inevitable inter-site differences in the presence of the producing organisms. The monitoring of TTX concentrations at specific sites also provides some insights into uptake and depuration rates, although the low sampling frequencies at some sites during this study prevented any thorough assessment. One site, where fortnightly sampling of hard clams was maintained throughout the summer period, showed indications of relatively slow uptake rates, with levels dropping to 25% of peak concentrations within a two-week period, followed by a further 50% drop in the subsequent two weeks. Similar depuration rates were also observed in native oysters. At the site found to contain the Pacific oyster sample with the highest TTX concentrations, a sampling issue resulted in a two-month gap between testing, preventing any assessment of depuration. Moreover, results obtained from one mussel harvesting area, showed an apparent rapid drop in TTX concentrations over a two-week period, prior to an increase in levels during the subsequent fortnight. Whilst there may be some indications of species-related differences in uptake and depuration patterns, overall, it is clear that a more systematic study of uptake and depuration rates is required, with a high sampling frequency, to better understand shellfish uptake dynamics for TTX toxins.

Eight different species of bivalve mollusc were assessed during this study. Pacific oysters, mussels, native oysters and hard clams were the four species found to contain TTXs above the method LOR, with the highest concentration recorded in Pacific oysters. It has to be noted that only one representative species was sampled in each monitoring site and parallel comparison between several species within one site was not possible. Our previous work has however highlighted higher TTX concentrations in Pacific oysters in comparison with mussels sampled at the same time and at the same site [16]. Whilst during 2014–2015, TTXs were only quantified at levels >20 µg/kg in mussels, hard clams and Pacific oysters, with higher concentrations quantified in native oysters during 2016. The year 2016 also saw the quantitation of higher concentrations of TTXs in hard clams in comparison to previous years, with a maximum level of 172 µg/kg TTXs being quantified during early July. The three bivalve species found not to contain detectable levels of TTXs were either harvested exclusively from Scotland (razors and surf clams) or were obtained off-shore as part of the wild *Petenidae* monitoring programme. As such, given the results highlighted here, accumulation of TTXs into any bivalve molluscs in these locations would not be expected. Consequently, from this study, some of the species-related differences are likely to reflect more the localized environmental situation, rather than specific differences in bivalve uptake mechanisms. Future work should therefore include the testing of multiple species from the same shellfish beds, ideally located in geographical areas likely to result in TTX uptake in molluscs. A comparison of our data with monitoring results obtained from Greece is difficult, because the study of [7] contained almost exclusively mussels. Out of the 48 sample results presented, 46 were mussels, with just two consisting of *Venus* clams (*Venus verrucosa*). One clam sample, however, showed total TTXs at a concentration of 195 µg/kg. This study therefore provides further confirmation for the potential accumulation of TTXs in clams, as well as mussels and oysters. The ubiquity of this toxin group across other seafood commodities therefore requires further elucidation.

The results from this study confirm the dominance of the parent TTX toxin, in comparison to other TTX analogues incorporated into the LC-MS/MS method. Interestingly, higher proportions of TTX analogues were quantified during 2015 in comparison to other years. During 2016, nearly all positive samples were found to contain 100% TTX, with no detectable presence of any other analogues. Just one sample was found to contain 5-deoxy TTX exclusively, albeit at a concentration close to the LOR (2.88 µg/kg). During 2015, however, 4-epi TTX, 5,6,11-trideoxy TTX and 4,9-anhydro TTX were all detected, again at low relative concentrations. Interestingly there were no differences in toxin profiles between shellfish species, with more positive results being required to examine this factor in greater depth. The method has been validated recently for mussels and oysters with full performance characteristics determined for the analogues TTX and 4,9-anhydro TTX. In the absence of certified reference materials, preliminary performance characteristics, including reproducibility have been determined for 4-epi TTX, 5,6,11-trideoxy TTX, 11-nor TTX-6-ol, and 5-deoxy TTX [18]. Consequently, there is the potential for other TTX analogues to be present which are not currently incorporated into the LC-MS/MS method. In this situation, there is the potential for total TTX levels to be under-estimated using the currently chemical detection method. The validated TTX method used in our laboratory can be run either as a stand-alone method for TTX and associated analogues, or alternatively run as a method for paralytic shellfish toxins (PST) plus the parent TTX [18]. This study therefore shows that to date, with one exception, the PST + TTX combined method would be suitable for continued screening of bivalve mollusc samples, given that results indicate the presence of TTX in all except one sample containing TTXs. The one sample found to contain only 5-deoxy TTX, showed a toxin concentration of 2.9 µg/kg, so well below any threshold of risk to the shellfish consumer. In Greece, TTX-positive samples were also reported as containing TTX predominantly, with lower relative concentrations of 4-epi TTX and 4,6-anhydro TTX in a small number of samples. This therefore provides further indications that a preliminary PST+TTX analysis would be a suitable approach for detecting TTXs in European mollusc samples, prior to a full quantitation using the stand alone TTX method. In addition, as more TTX analogues become available, either as reference materials, or in naturally contaminated tissue materials, the TTX method [18] can be extended to incorporate additional TTX analytes.

As with the noted prevalence of regulated marine toxins such as PST in the UK [23], there is evidence from this study for the highest levels of TTX accumulation during the warmer summer months. The highest TTX concentrations were quantified in samples harvested between June and August each year, relating to the time of year when seawater temperatures are higher. TTX detection was not confined to these three months exclusively, however, with the quantitation of one sample at 19.9 µg/kg in January 2016, and with the detection of TTX in other samples outside of the warmer season. This confirms our earlier work, where TTX was detected in mussel and oysters samples throughout the year [16]. Consequently, any future risk assessment should not exclude sampling during the winter, spring and autumn seasons, until more systematic data have been collected and assessed.

### 3.2. Potential Causative Factors

The areas of the UK coastline found to contain TTX-contaminated shellfish, show the south coast of England to be most at risk from accumulation of this group of toxins in bivalve molluscs. This is the area of the coast with the largest number of sunlight hours and the highest mean air temperatures through the year [24]. The seawater temperature data, collected from harvesting sites during official control sampling of molluscs, illustrated an indicative threshold above which TTX-positive samples were more likely to be found. With one exception, all samples containing total TTX concentrations above 10 µg/kg were collected in waters above 15 °C. The one occurrence of TTX above 20 µg/kg in Scotland was sampled during a sustained sea surface temperature anomaly during July 2014 in the North Sea, with temperatures more than 3 °C higher than expected [25]. This TTX-positive sample notably corresponded temporally with a significant *Vibrio* outbreak observed in the Baltic Sea [26].

Following the categorization of shellfish harvesting areas into three zones relating to water depth, results indicated that the majority (93%) of TTX-positive shellfish were obtained from either inter-tidal or shallow water (<5 m depth) sites. In addition, 82% of TTX-positive samples were located in estuarine areas, containing lower salinity levels than those further out to sea. Whilst no specific depth and salinity measurements are taken routinely during official control sampling activities, this categorization has indicated a clear preference for TTX accumulation to occur in shallow water, lower salinity, estuarine environments, in direct contrast to deeper waters in the open sea, or in riverine environments. Future work would incorporate a more precise assessment of these factors, utilizing both water depth and salinity determination.

These findings fit with the hypothesis that bacteria such as *Vibrio* species, are responsible for production of TTX, ultimately resulting in toxin accumulation within bivalve molluscs, as highlighted by other studies since 1986 e.g., [10,11,13,26,27,28,29]. Indeed, there are recent indications that 31 genera of bacteria have been isolated which have been found to produce TTX [5], with the majority belonging to the Gammaproteobacteria class, including the genera *Aeromonas, Alteromonas, Pseudomonas, Shewanella* and *Vibrio*, as well as Alphaproteobacteria and Betaproteobacteria classes. Our previous work has clearly shown both the isolation of *Vibrio parahaemolyticus* in UK waters and shellfish [30] over the past decade and the joint presence of both TTXs and *Vibrio* sp. in mussels and Pacific oysters harvested from two sites in the south of England during 2013–2014 [16]. Furthermore, strong evidence was also shown for the detection of TTX in *Vibrio* cultures isolated from bivalve molluscs [16]. In addition, the recent work of [8] has confirmed the presence of *Vibrio* and *Pseudomonas* species in Greek mussels, also shown to contain TTXs. Furthermore, a recent review has summarised that *Vibrio* species comprise more than 30% of the currently isolated TTX-producing bacterial strains [5]. Since our initial findings in England, there have been very few data gathered in the UK on *Vibrio* occurrence, so there is no way of knowing about overall prevalence of *Vibrio* across the country, until more work has been conducted. The findings in this study do however add further weight to the bacterial origin of these toxins, given that sites at higher risk of *Vibrio* are likely to be shallow, lower salinity, estuarine waters, above 15 °C [30]. Changes in seawater temperatures will influence the growth rates of marine microorganisms. In particular, higher temperatures, increased precipitation and subsequent reductions in sea surface salinity is likely to impact a range of marine organisms, including *Vibrio* spp. [31]. Numerous laboratory and epidemiological studies have shown that temperatures above this temperature threshold increase the risks associated with these bacteria [5,32,33,34,35]. It is now well recognised that the start of the warm seasons has become earlier in a significant number of temperate coastal zones, with an increase in the number of hot days each year particularly evident at high latitudes [33] through the North Sea and English Channel [36]. Certainly, recent climatic data indicate that the region of GB discussed in this study (the English Channel) has warmed recently [36], with sea-surface temperatures around the UK coast rising over the past 30 years alone by over 0.7 °C [37] Consequently, with other strong evidence for the continued warming of the seas around the UK [24], the likelihood of continued if not increasing risks from marine biotoxins [38,39,40,41] including TTXs in England should not be discounted.

The work of [7] proposed the link between *Prorocentrum cordatum* (*minimum*) and Tetrodotoxin in shellfish from Greece. The link was evident initially from the occurrence of both at the same period of time, with samples containing higher concentrations of TTXs linked to the prevalence of *P. cordatum* blooms, and those with lower TTX concentrations sampled when blooms were not recorded. More recently, [8], described the production of TTX-like compounds with similar mass spectral behaviour to TTX analogues by bacteria present in *P. cordatum*. Results from this study however, do not show any correlation between the presence of *P. cordatum* in the water column and TTXs in shellfish flesh. At times of the year, *P. cordatum* can reach very high levels in England, exceeding cell densities of 1 × 10^6^ cells/L. From our results to date, sites exhibiting such densities have not coincided with shellfish accumulating high levels of TTXs as proposed by [7,8]. Similarly, *P. cordatum* is extremely common in water samples taken throughout Scotland, also reaching very high cell densities (>4 × 10^6^ cells/L) [42], whereas TTX was, with only a minor exception, not present in Scottish shellfish. Clearly, whilst phytoplankton species such as *P. cordatum* may provide a suitable environment for TTX-producing bacteria in other regions of Europe such as Greece, there is no evidence for this to be occurring in recent years within UK waters. Nevertheless, until the uptake mechanisms for TTX in molluscs has been fully elucidated, this exposure route should not be completely discounted. Future work to determine the potential mechanisms for TTX production and uptake should include not just the analysis of bacterial culture, but also environmental water samples from TTX positive shellfish beds and cultured samples of a range of marine phytoplankton, including *P. cordatum*.

## 4. Materials and Methods

### 4.1. Reagents and Chemicals

Instrument solvents were LC-MS-grade (Fisher Optima, Thermo Fisher, Hemel Hempstead, UK) and all chemicals were LC-MS reagent grade where possible. Sample preparation and SPE reagents were HPLC grade. TTX CRM was obtained from Cifga (Lugo, Spain). A freeze-dried naturally contaminated tissue of the sea slug *Pleurobranchaea maculata* containing extremely high concentrations of TTX and TTX analogues was purchased from Cawthron Natural Compounds (CNC; Nelson, New Zealand).

A TTX stock standard solution was prepared from the Cifga CRM and used to prepare working standards for quantitation by external calibration. The CRM contains 25.1 ± 1.3 µg/g TTX, together with 2.99 ± 0.21 µg/g 4,9-anhydro TTX and trace levels of 4-epi-TTX, 11-deoxy TTX, 11-nor TTX-6-ol and 11-trideoxy TTX. One Cifga TTX CRM ampoule was opened and the contents diluted by a factor of ten using deionised water. An additional stock at 100× dilution was also prepared using water as the diluent. This solution was used for preparation of LC-MS/MS calibration standards at six concentration levels by diluting in 80% acetonitrile (MeCN) with 0.25% acetic acid. Fresh solutions were prepared weekly and once prepared stored in a refrigerated autosampler. Due to the low concentrations of the other TTX analogues present in the CRM, quantitation was performed using TTX. Analysis of concentrated CRM solution diluted by a factor of 10, enabled the detection of the minor analogues which enabled the confirmation of these analogues in QC samples, thereby facilitating confirmation in unknown samples.

### 4.2. Shellfish Samples

Following on from the first detection of TTXs in Southern England during 2013–2014 [16], a range of bivalve mollusc samples were subsequently subjected to testing at Cefas for TTXs. Shellfish samples analysed during this study were obtained primarily through the official control (OC) monitoring programs of the United Kingdom, including Scotland, Northern Ireland, England and Wales from classified shellfish production areas (Figure S2). These were sourced between 2014 and 2016 inclusive and included a variety of species such as common mussels (*Mytilus edulis*), Pacific oysters (*Crassostrea gigas*), native oysters (*Ostrea edulis*), hard clams (*Mercenaria mercenaria*), cockles (*Cerastoderma edule*), razors (*Ensis* spp.) and surf clams (*Spisula solida*). Shellfish samples were collected by either dedicated shellfish sampling officers, or by Local Authorities. Seawater temperatures were also measured during sample collection where possible and practical. In addition, king scallops (*Pecten maximus*) were received through the UK official control monitoring programme for verification of wild *Pectenidae*.

- Year 2014 samples: 603 OC samples and 13 scallop verification samples: Scotland 476 (6 scallop), England 120 (7 scallop), and Wales 20.
- Year 2015 samples: 408 OC samples and 48 scallop verification samples: Scotland 194 (4 scallop), England 254 (44 scallop), Wales 8.
- Year 2016 samples: 57 OC samples from Northern Ireland and 154 OC samples from selected sites in Southern England.

Shellfish samples were extracted fresh for PST toxin analysis using 1% acetic acid. The extracts were stored frozen (<−15 °C) until required for TTX clean-up and HILIC-MS/MS analysis. A sample was created consisting of an acetic acid extract of mussel mixed with very low proportions of the sea slug acidic extract (<0.1% *v*/*v*), which was used as a chromatographic retention time marker for TTX and other TTX analogues. The sea slug sample contained high concentrations of TTX, together with high levels of 4-epi-TTX, 5,6,11-trideoxy TTX, 11-nor TTX-6-ol, 5-deoxy TTX, and 4,9-anhydro TTX. Confirmation of these additional analogues was made using expected MRMs and comparison with the TTX analogues present in the Cifga CRM solution. These also enabled expected ion ratios to be established between primary and secondary MRMs, facilitating detection and quantitation of these analogues in unknown samples. Two laboratory reference materials (LRM) were also prepared in-house and used as positive control materials, one mussel and one Pacific oyster. Shellfish tissues from both species were fortified (<0.1% *w*/*w*) with spikes of raw acetic acid extracts of the sea slug RM containing very high concentrations of TTX analogues. The bulk spiked mussel and oyster tissues were homogenised and aliquoted into 5.0 ± 0.1 g sub-samples, before being stored frozen (<−15 °C) until required for testing.

### 4.3. Sample Preparation and Toxin Analysis

Shellfish samples were extracted using AOAC2005.06 double extraction method as part of the routine monitoring for PSP as described by [43]. The crude extracts were subsequently cleaned-up using conditioned Supelclean Envi-carb solid phase extraction cartridges based on [44,45], prior to dilution in acetonitrile prior to LC-MS/MS analysis as described by the single-laboratory validated TTX method of [18].

HILIC-MS/MS using a Waters (Manchester, UK) Acquity UPLC I-Class coupled to a Xevo TQ-S tandem quadrupole mass spectrometer (MS/MS) was conducted exactly as described by [18]. Each instrumental sequence started with a start-up inlet and finished with shutdown inlet methods as detailed by [18,44]. MRM transitions are summarised in [18]. The MS method involves the direct quantitation of TTX against an external TTX calibration. The additional analogues (4-epi-TTX, 5,6,11-trideoxy TTX; 11-nor TTX-6-ol; 4,9-anhydro TTX; 5-deoxy TTX/11-deoxy TTX) were incorporated into the method, with semi-quantitation conducted using the parent TTX calibration. Confirmation of the detection of TTX analogues was performed using the comparison of primary and secondary MRM peaks against those present in the analytical standard and the quality control samples. The ratio between the two MRMs was also used for confirmatory purposes. The Limit of Detection (LOD), Quantitation (LOQ) and Reporting (LOR) were presented previously by [18]. During this study, however, the LOQ for each analogue was found to be lower than 2 µg/kg, with the LOQ for TTX < 1 µg/kg. Consequently, the LOR used in this study was taken as 2 µg/kg. No Toxicity Equivalence Factors (TEFs) were applied to calculations of TTX analogues, given the current absence of any formal published recommendations and concentration data were not adjusted for recovery.

### 4.4. Phytoplankton Collection and Analysis

Water samples were collected and sent to the laboratory by trained sampling officers from local authorities. The aim of the water collection method is to obtain samples which are representative of the algal community in the water body being sampled. Each water sample is therefore taken as close to the shellfish bed as possible and as close to the location from where shellfish samples for flesh testing are taken. The sampling method used is dependent on the depth of water at the site but all collectors were asked to collect water samples at high tide (±1 h), particularly at inshore sites. Tube samplers [46] are provided to collectors who have access to boats, or where piers and jetties were sufficiently close to the sampling points to allow a depth integrated sample to be taken. However, it was recognised that their use was not always practical in shallow, coastal areas and a homogenised sample, collected from three depths (near bottom, midwater and near surface) using a pole sampler, was recommended as a preferential alternative to sampling surface water with a bucket. A surface bucket sample was, however, collected where the depth of water precluded the other methods. The water collected was homogenised gently in a bucket and poured into a 500 mL Nalgene bottle. Each sample was preserved with the addition of 2 mL of acidified Lugol’s Iodine and posted to the Cefas plankton laboratory for analysis. On arrival at the laboratory, the samples were initially set-up in 25 mL Utermöhl chambers and left to settle following the procedures set out in International Oceanographic Commission Guide 55 [47]. After a minimum of three hours, each sample was given a preliminary examination. If the base plate of the chamber was obscured by too much sediment, or too many cells then an additional sub-sample was set up in a 10 mL or 5 mL Utermöhl chamber. All samples were then allowed to settle for a minimum of 12 h before identification and enumeration of the micro-algal cells using high-power, inverted microscopes.

### 4.5. Water Temperature Monitoring

Shellfish sampling personnel collecting shellfish for official control monitoring also record water temperatures at the time of collection from the same site. Various methods are used throughout the UK for measuring temperature, depending on the geographical area and available resources, with the majority utilising handheld and calibrated electronic temperature monitoring probes. When the shellfish were collected from water the temperature of the surrounding seawater was measured and recorded. If the shellfish were not submerged in water, for instances of inter-tidal shellfish which are sampled dry, the temperature of the bagged shellfish was taken and recorded instead.

## Figures and Tables

**Table 1:** Summary of results obtained following HILIC-MS/MS analysis of TTXs in bivalve molluscs from OC monitoring sites in England, Wales, Scotland and Northern Ireland.

Country	Year	Samples Tested	Results > LOR ^1^	Results > 20 µg/kg	Maximum Concentration µg/kg	Dates for Positives (>LOR)
England	2014	113	1 (0.9%)	0	6	na
	2015	210	21 (10%)	6 (3%)	253	July–August
	2016	154	33 (21%)	8 (6%)	93	January–July
Wales	2014	20	0	0	na	na
	2015	8	0	0	na	na
	2016	0	0	0	na	na
Scotland	2014	470	2 (0.4%)	1 (0.2%)	26	July
	2015	190	0	0	na	na
	2016	0	0	0	na	na
N. Ireland	2014	0	0	0	na	na
	2015	0	0	0	na	na
	2016	57	0	0	na	na

na = not applicable. ^1^ LOR = 2 µg/kg.

**Table 2:** Summary of the number of TTX-positive samples, above LOR and 20 µg/kg threshold, in relation to ranges of seawater temperature.

	Temperature	0–4 °C	5–9 °C	10–14 °C	15–19 °C	20–24 °C
England	Total	0	25	86	185	26
	>LOR	0	3 (12%)	2 (2%)	39 (21%)	3 (12%)
	>20 µg/kg	0	0	0	12 (7%)	2 (8%)
Scotland and Wales	Total	3	19	184	51	0
	>LOR	0	0	0	0	0
	>20 µg/kg	0	0	0	0	0

**Table 3:** Summary of site categorization in terms of (a) water depth and (b) salinity setting.

All Sites	0–5 m	5–20 m	>20 m	River	Estuary	Open
55	51 (93%)	4 (7%)	0	6 (11%)	45 (82%)	4 (7%)

## Supplementary Materials

The following are available online at www.mdpi.com/1660-3397/15/9/277/s1. Figure S1: Structures of selected Tetrodotoxin analogues, Figure S2: Map of UK showing location of shellfish samples taken during this study (blue dots).
