# Supplementary material for: Detection of Tetrodotoxin Shellfish Poisoning (TSP) Toxins and Causative Factors in Bivalve Molluscs from the UK
Source: Mar Drugs. 2017 Aug 30;15(9):277. doi: 10.3390/md15090277 (PMC5618416; doi:10.3390/md15090277)

Figure S1. Structures of selected Tetrodotoxin analogues

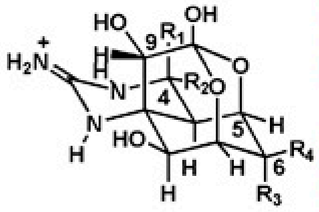

Analogue	R_1	R_2	R_3	R_4
TTX	H	OH	OH	CH_2OH
4-*epi*-TTX	OH	H	OH	CH_2OH
11-deoxy TTX	H	OH	OH	CH_3
6,11-dideoxy TTX	H	OH	H	CH_3
11-norTTX-6(S)-ol	H	OH	OH	H
11-norTTX-6(R)-ol	H	OH	H	OH

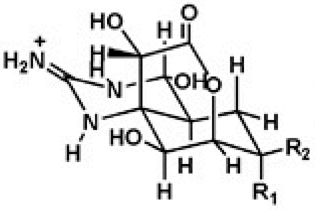

Analogue	R_1	R_2
5-deoxy TTX	OH	CH_2OH
5,6,11-trideoxy TTX	H	CH_3

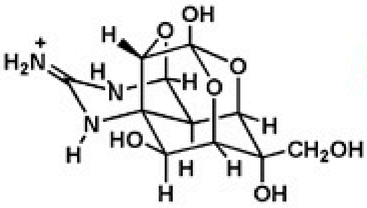

4,9-anhydro TTX

Figure S2. Map of UK showing location of shellfish samples taken during this study (blue dots)

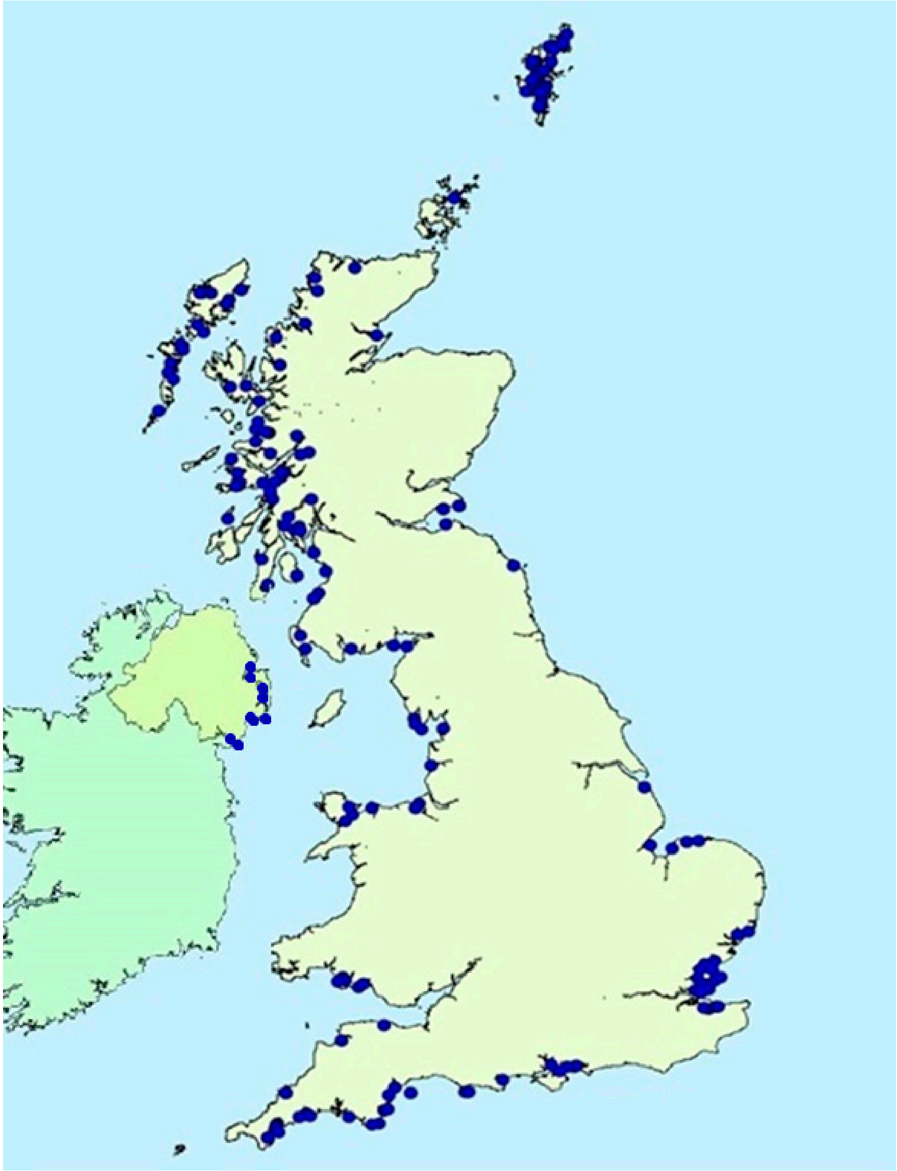

Supplement: Supplementary file 1 [file marinedrugs-15-00277-s001.pdf]
